# SAASNets: Shared attention aggregation Siamese networks for building change detection in multispectral remote sensing

**DOI:** 10.1371/journal.pone.0306755

**Published:** 2025-01-30

**Authors:** Shuai Pang, Chaochao You, Min Zhang, Baojie Zhang, Liyou Wang, Xiaolong Shi, Yu Sun

**Affiliations:** 1 Shandong University of Aeronautics, Binzhou, China; 2 Zhanhua District Power Supply Company, Binzhou, China; University of Thessaly, School of Engineering, GREECE

## Abstract

Interfered by external factors, the receptive field limits the traditional CNN multispectral remote sensing building change detection method. It is difficult to obtain detailed building changes entirely, and redundant information is reused in the encoding stage, which reduces the feature representation and detection performance. To address these limitations, we design a Siamese network of shared attention aggregation to learn the detailed semantics of buildings in multispectral remote sensing images. On the one hand, a special attention embedding module is introduced into each subspace of the feature extractor to promote the interaction between multi-scale local features and enhance the representation of global features. On the other hand, a highly efficient channel and position multi-head attention module is added to the Siamese features to encode position details while sharing channel information. In addition, adopting a feature aggregation module with a residual strategy to fuse the features of different stages of the Siamese network is beneficial for detecting different scales and irregular object buildings. Finally, experimental results on LEVIR-CD and CDD datasets show that designed SAASNets have better accuracy and robustness.

## Introduction

As one of the many data sources for observing the earth’s surface, multispectral remote sensing images are widely used in many tasks [1–3], such as military, ecological, and ocean monitoring. Building change detection plays a vital role in urban land resource monitoring. Its primary purpose is to monitor the changed areas by comparing the differences of the same scene in different periods. With the rapid development of the economy and the acceleration of urbanization, buildings, as an essential part of the city, symbolize urbanization and a meaningful way to shape the city’s style and culture. A real-time grasp of the changes in urban buildings is an essential data basis for improving urban governance capabilities and solving problems such as “big city diseases.” However, urban buildings are relatively complex and have many categories, which brings enormous challenges to the statistics of changed areas. The early architectural change detection methods focused on pixel-level differential statistical methods [4]. These simple machine learning methods use individual pixels as perception units, which are difficult to use on a large scale. The detection accuracy is low. At the same time, manual participation in feature design and screening is required. Expert experience seriously affects the detection effect. Deep learning technology achieves automation by learning discriminative information from images, texts, and voices [5–7]. It has been successfully applied in many fields and provides a new way of thinking.

In recent years, convolution-based neural networks have been widely used in building change detection tasks [8, 9], relying on their powerful feature self-extraction capabilities. These methods use the activation state of neurons to obtain in-depth spatial features of buildings, avoiding manual participation in design features. However, due to the limitation of the acceptance field, it isn’t easy to fully obtain detailed information about the building target, and the timing interaction information between different timing remote sensing images is ignored. Recurrent neural networks [10, 11] can model the temporal semantics of architectural objects. The interpretability of neurons could be improved, and the network is prone to problems such as overfitting, gradient disappearance, and gradient explosion. At the same time, it cannot effectively establish long-distance dependencies. There is still much room for improvement in detection accuracy. Applying LSTM [12, 13] to multispectral remote sensing building detection tasks is beneficial to establishing long-term dependencies and effectively solves problems such as gradient disappearance. However, increasing network parameters decreases detection efficiency, and it is difficult to apply in real-time change detection tasks. Due to the interference of external environmental factors such as imaging angle, weather, and illumination and the complex semantic content of multispectral remote sensing images, traditional deep learning methods find it difficult to detect changes in buildings at different scales in images fully. In addition, multispectral remote sensing images of the same scene but different time phases contain rich spatial information and interaction between channel information. These algorithms do not fully use channel information and ignore the information interaction between channels. Although some change detection methods refine the feature maps by introducing an attention mechanism after the feature extractor, they still ignore the refinement of the information flow in the encoding stage.

To address the above issues, we design a Shared Attention Aggregation Siamese Network (SAASNets) to capture rich spatial details and temporal semantics of architectural objects in multispectral remote sensing images.

The main contributions of this study are as follows:

1. Each feature extraction module in the encoding stage is divided into multiple subspaces to capture the building’s local spatial details entirely. A particular attention module is embedded in each subspace to refine the local features and focus on architectural object context and global semantics in multispectral remote-sensing images. A weighted loss function is developed to improve semantic representation performance by individually supervising each feature extraction stage.
2. An efficient channel and position multi-head attention module are designed. To fully use the channel information of two sets of images at different times and in the same scene, strengthen the interaction between channel information and simultaneously model the sensitive positions from dependencies. In addition, a residual strategy attention feature aggregation module is designed to fuse the conjoined features at different stages so that the network can better model architectural objects of different scales and effectively perceive the details of irregular object buildings. Finally, it was demonstrated on various baseline datasets that all achieved the best performance.

The remainder of the paper is organized as follows. Related work is presented in Section 1. SAASNets for multispectral remote sensing building change detection will be described in detail in Section 1. Experimental results and analysis are provided in Section 1. Section 1 presents conclusions and further research plans.

## Related works

Early building change detection focused on simple machine learning methods. For example, Zhang et al. [14] designed a classic local binary algorithm for building detection in very high spatial resolution (VHR) images for high-resolution remote sensing images with histogram equalization and bilateral filtering to enhance the contrast and building edges. The low-density feature map is obtained through the classic local binary pattern (LBP) algorithm, and then the ground features are divided into objects by mean shift based on the feature map. To facilitate land management and urban planning, Xi et al. [15] proposed a morphological method to detect building changes on multi-time camera light-borne detection and ranging data, that is, through irregular triangular network filters and support vector machines and different time The acquired two sets of point clouds divide the three types of land use into ground, buildings, and vegetation. And apply morphological algorithms to distinguish changing buildings and trees, reducing recognition errors. Considering spectral heterogeneity and complexity, Wang et al. [16] designed a refined new building area detection method based on generalized machine learning. Zong et al. [17] proposed an irregular Markov random field model to detect building changes from multi-temporal high-resolution aerial images. The method analyzes building changes in the spatial domain and explores contextual relations to achieve robust detection results. Although these methods effectively improve the accuracy of building change detection in remote sensing images, they use handcrafted features and have low automation efficiency. At the same time, it can be challenging to apply on a large scale due to manual participation in feature design and screening.

Deep learning technology has also been widely used in building change detection tasks. For example, Saha et al. [18] considered that multi-temporal SAR images are complex and have high spatial correlation issues, using pairs of unmarked SAR and optical images to train using cycle consistency Generative Adversarial Networks (CycleGAN) sub-optimal tasks of transcoding SAR images to optical images and detecting architectural changes. Using the semantic segmentation of buildings as an auxiliary information source for fine-grained change detection, Sun et al. [19] designed a deep multi-task learning framework for detecting building changes from VHR images, which employs an encoder-decoder architecture that simultaneously addresses the main task of change detection and the auxiliary task of semantic segmentation. Jiang et al. [20] proposed an end-to-end network guided by pyramidal feature attention, which uses the convolutional neural network in the pyramid to capture possible changes and emphasizes the correlation and saliency between input feature pairs by introducing a global co-attention mechanism. Feature importance, meanwhile, utilizes various attention mechanisms to aggregate features from low-level and co-attention layers, effectively improving the long-term dependencies of features. To solve the problem of insufficient data for change detection tasks, Chen et al. [21] proposed an instance-level change enhancement method by utilizing generative adversarial training to generate bitemporal images containing changes involving a large number of diverse buildings and transferring these data aware of contextual details of architectural objects into a simple and effective change detection network. To better obtain the high-frequency patterns under the deep learning pipeline, Zheng et al. [22] proposed a high-frequency attention-guided Siamese network, which enhances the high-frequency information of buildings through the built-in high-frequency attention block and promotes the network to detect better edge details of changing buildings to improve detection performance. Based on a fully convolutional network and an attention mechanism, Song et al. [23] proposed an attention-guided end-to-end change detection network that learns feature representations to enhance change information and utilizes spatial attention and channel attention to achieve accuracy. Among them, the spatial attention module facilitates the discrimination between varying objects and backgrounds by adding the learned spatial attention to deep features. The channel attention-guided interference filtering unit and the porous spatial pyramid pooling module enhance the representation of multi-level features and multi-scale context, respectively, and finally improve the accuracy of building change detection. Although these methods utilize multi-scale information and attention features, they ignore the correlation between buildings and surrounding tissues and often use irrelevant information in the feature extraction process, which reduces the representation performance of salient features. Edge details and long-term dependencies of objects. Bai et al. [24] proposed an edge-guided recurrent convolutional neural network for architectural change detection, which mainly combines discriminative information and edge structure into one framework to generate more accurate architectural boundaries and train Siamese convolutions. The integrated neural network extracts main multi-level features from multi-temporal images simultaneously. Xue et al. [25], considering the heterogeneity of remote sensing images and large-scale building changes, propose a multi-branch network structure to fuse semantic information of building changes at different levels. In this network, two auxiliary branches guide the semantic information of buildings under different time series, while the main branch can incorporate the change information. Considering the inter-class similarity and intra-class difference of building changes in complex built-up areas, Liu et al. [26] proposed an end-to-end PA-Former method for building change detection, that is, by learning a priori perception Transformer, the prior Extraction and context fusion are combined. To enhance the model’s ability to resist spurious changes and improve detection accuracy, Zheng et al. [27] proposed a multi-task differential enhanced Siamese network for architectural change detection in high-resolution remote sensing images. The network improves its performance by adding semantic constraints. Feature extraction ability, and effectively use features while improving its recognition performance, at the same time, using feature difference enhancement module to measure the difference and similarity of buildings in two-temporal images. Li X et al. [28] considered that Deeplabv3+ easily ignores small-sized targets and is usually unable to identify precise segmentation boundaries. They proposed a semantic segmentation algorithm for UAV remote sensing images based on edge feature fusion and multi-level upsampling combined with Deeplabv3+ (EMNet, although this network improves the representation of edge effects, the parameter setting of this network is more complicated after adding edge loss. Coupland K et al. [29] summarized the tree canopy cover (TCC) of lidar and aerial photo data into an analysis polygon of 0.05% ha to compare the tree canopy cover (TCC) of the two different data types.

## Our proposed method

This section first presents the basic workflow of the proposed SAASNets multispectral remote sensing building change detection framework. Second, the multi-scale feature extraction module (LAFEM) embedded with special attention is introduced in detail, and the channel and position multi-head attention module (CPMA) is elaborated. Finally, the Attention Siamese Feature Aggregation Module (RASAM) of the residual strategy is described, and the related calculation of the weighted loss function is provided.

### Overview

*X*_*A*_, *X*_*B*_ denotes different time-series remote sensing building images. $\left\{f_{X_{A}}^{\left\{0,1,2,3,4\right\}},f_{X_{B}}^{\left\{0,1,2,3,4\right\}}\right\}$ represent multi-scale feature information at different levels, respectively. $f_{X_{A}^{s}}$ represents the multi-scale features of different time series images; $f_{X_{A}}^{s,c}$ represents multi-scale. Fcc represents the fused feature information. *CLM* stands for classification module. ‘Outputs’ represents output results. $f_{X_{A}}$ and $f_{X_{B}}$ represent the conjoined features of the final output of different timing branches.

**Fig 1:**
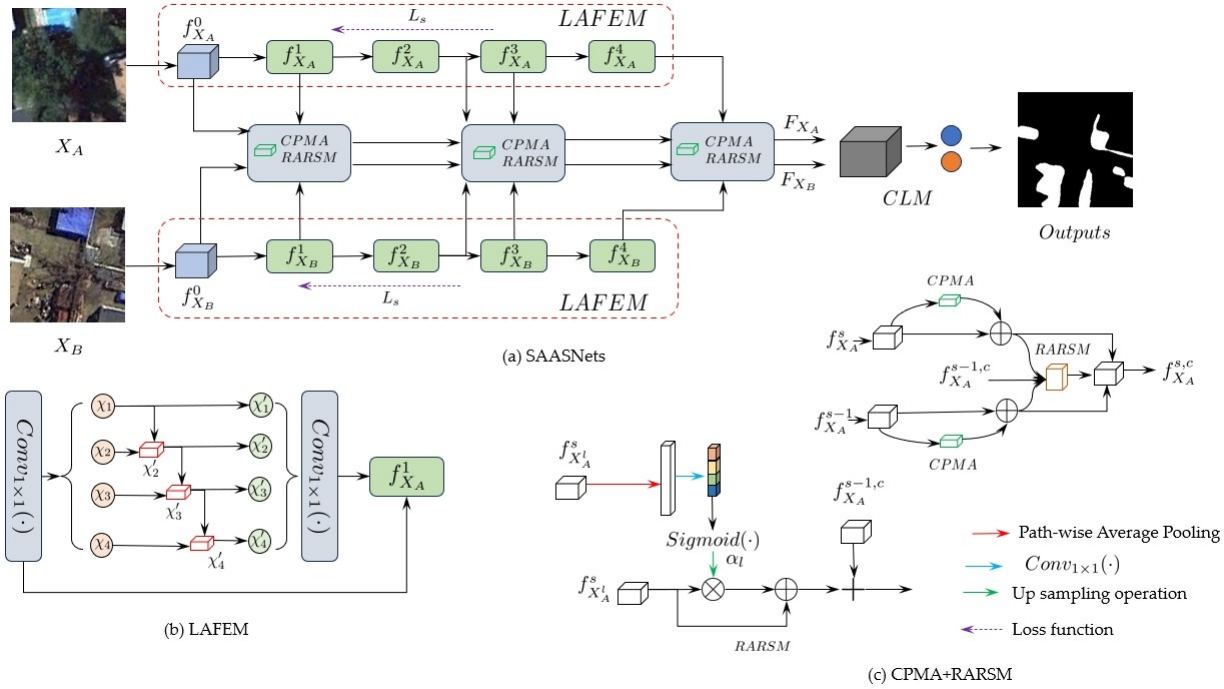
(a) denotes our proposed SAASNets remote sensing building change detection framework. (b) denotes Feature Extraction Module with Special Attention Embedding (LAFEM). (c) denotes channel position multi-head attention module (CPMA) and attention feature fusion module with residual strategy (RASAM).

The architecture change detection framework of the proposed SAASNets multispectral remote sensing images is shown in Fig 1. The framework mainly includes the initial feature extraction module (LAFEM) of special attention embedding, the information fusion module (CPMA) of channel position multi-head attention [30–32], the conjoined feature aggregation module (RASAM) of residual strategy and the decoding classification module, etc. Among them, the initial feature extraction module (LAFEM) of special attention embedding [33] aims to obtain architectural details of different scales by dividing subspaces. It uses particular attention components to refine this feature information to improve multi-scale local features and the Representation power of salient information in global semantics. The information fusion module (CPMA) of channel position multi-head attention mainly models channel and position information, obtains the correlation between channel information, establishes dependence between position information, and further reduces the use of redundant information. As well as establishing interactions between local and global features to improve the semantic representation performance of the global context. The residual strategy’s siamese feature aggregation module (RASAM) mainly aggregates local features and global semantics at different stages and scales. It forms a complementarity between them to enhance the representation of features on the details of building objects. Finally, these Siamese features are decoded in the decoding and classification module, and the classification of the changed and non-changed areas is realized.

### LAFEM

Multi-scale features have been widely used in change detection tasks and are beneficial to improve object representation and detection performance. However, it is difficult to fully obtain the deep spatial details of the target by using the traditional residual convolutional network. At the same time, redundant information is used many times in the process of information flow transmission, which reduces the representation of salient features and thus affects detection accuracy. Therefore, we designed an initial feature extraction module embedded with a unique attention mechanism; that is, the feature extraction module is divided into multiple subspaces and convolution and attention are combined in the subspaces to reduce redundant information and highlight the representation of salient features. It enhances the representation of global semantics while capturing local details. The specific steps are as follows.

**Step 1**. Assume that the input multispectral remote sensing image pair is $X=\{X_{A},X_{B}|\in R^{H\times W\times C}\}$, where *H*, *W* and *C* represent the images height, width, and channel dimensions, respectively. it input into a 7×7 large-scale convolution for operation, and use maximum pooling for feature compression. The operation is as shown in the equation.

$$\begin{array}{c} f_{X_{A}}^{0}=MaxPooling\left(Conv_{7\times 7}\left(X_{A}\right)\right)\in R^{H\times W\times C} \end{array}$$ (1)

Where, $f_{X_{A}}^{0}=f_{X_{B}}^{0}$, *MaxPooling*(⋅) represents the maximum pooling operation. *Conv*_7×7_(⋅) represents a 7 × 7 convolution operation.

**Step 2**. Input $f_{X_{A}}^{0}$ into a 1 × 1 convolutional layers, perform feature extrusion and divide it into four subspaces for convolution and attention operations to obtain a refined attention feature map. The operation is as shown in the equation.

$$\begin{array}{c} \chi_{k}=Conv_{1\times 1}\left(f_{X_{A}}^{0}\right)\in R^{H\times W\times C},k=1,2,3,4 \end{array}$$ (2)

$$\chi_{k}^{\prime }=Conv_{3\times 3}\left(\alpha_{k}^{\prime }\cdot \left(\chi_{k}+\chi_{k-1}^{\prime }\right)\right),k=2,3,4$$ (3)

Where, when *k* = 1 indicates the $\chi_{k}^{\prime }=\chi_{k}$, *Conv*_3×3(⋅)_, *Conv*_1×1_(⋅)represent 3 × 3 and 1 × 1 convolution operations, respectively. *α*_*k*_ represents local attention embedding feature maps at different levels. *α*_*k*_ is shown in the equation.

$$\begin{array}{c} \begin{array}{cc} \alpha_{k}^{\prime } & =LAE(\chi_{k},\chi_{k-1}^{\prime })\\ & =\frac{1}{2}\cdot \left(\chi_{k}+\alpha \cdot \chi_{k}+\chi_{k-1}^{\prime }+\alpha \cdot \chi_{k-1}^{\prime }\right) \end{array} \end{array}$$ (4)

Where, *LAE*(⋅) denotes a special local attention embedding module. *α* represents the attention coefficient, and the equation shows the calculation.

$$\begin{array}{c} \alpha =\zeta_{u}^{↑}\left(\sigma_{1}\left(\zeta_{l}\cdot \sigma_{2}\left(Conv_{1,r}^{↓}\cdot \zeta_{h}\right)\right)\right) \end{array}$$ (5)

Where, $\zeta_{u}^{↑}$ represents an upsampling operation. *σ*_1_, *σ*_2_ represents the activation function sigma and *PReLu*, respectively. $Conv_{1,r}^{↓}$ represents the dimensionality reduction convolution operation with a reduction rate of 1 × 1.

**Step 3**. Fusion this subspace information and input 1 × 1 convolutional layer to generate a feature map $\left\{f_{X_{A}}^{s},f_{X_{B}}^{s}|s=1,2,3,4\right\}$ with spatial local and global details; the calculation is shown in the equation.

$$\begin{array}{c} f_{X_{A}}^{s}=Conv_{1\times 1}\left(\sum_{k=1}^{K}\chi_{k}^{\prime }\right),k=1,2,3,4 \end{array}$$ (6)

Where, *s* represents different feature scales. *k* represents the number of subspaces. $f_{X_{A}}^{s}$ represents the output characteristics of the remote sensing image *X*_*A*_ before the change in the *s*^*th*^ stage, and $f_{X_{B}}^{s}$ represents the output characteristics of the remote sensing image *X*_*B*_ after the change in the *s*^*th*^ stage.

In conclusion, adopting our designed feature extraction module benefits finer-grained feature representation, highlighting salient feature representation while reducing redundant information.

### CPMA and RASAM

Contextual semantic information is crucial for building change detection. Although the traditional global pooling operation can effectively aggregate contextual information, the image information has significant semantic ambiguity due to the complex content of remote sensing images. Therefore, to alleviate these problems, we design a channel- and position-based multi-head attention module, aiming at modeling the channel-correlation and position-dependence of architectural objects in remote sensing images to take full advantage of patch attention to enhance contextual information embeddings and improve the semantic representation of channel salient features. The feature map of multi-head attention is shown in equation.

$$\begin{array}{cc} f_{X_{A}}^{\prime s} & =\Phi_{CPMA}⊕f_{X_{A}}^{s} \end{array}$$ (7)

$$\phi_{CPMA}=Concat\left(\phi_{1}^{*},\phi_{2}^{*},\phi_{3}^{*}\right)\cdot \omega^{o}$$ (8)

$$\phi_{t}^{*}=f_{ca}⊕f_{sa}$$ (9)

Where *ω*^*o*^ denotes the matrix weights of the last linear layer. *Concat*(⋅) stands for feature connection. *f*_*ca*_ and *f*_*sa*_ denote the channel attention feature map and location attention map, respectively.

Each high-level feature channel map can be viewed as a response to architectural goals, and the semantic responses are interrelated. By exploiting the correlation between channel maps, the interdependent feature maps can be enhanced, and the feature representation with specific semantics can be improved to distinguish better the changes of buildings in different time-series remote sensing images. Therefore, we construct a channel attention module to establish the relationship between channels. In addition, compared with the spatial position attention, no convolution operation is used in the channel attention module to obtain new features. We reconstruct the new feature *F*′ on the feature *F*. Then, the matrix multiplication of the reconstructed feature *F*′ and its transpose is performed, and softmax obtains the channel attention feature *f*_*ca*_ of the building. *f*_*ca*_ is shown in equation.

$$\begin{array}{c} f_{ca_{j}}=\gamma \sum_{i=1}^{C}\left(\frac{exp\left(F_{i}\right)\cdot F_{j}}{\sum_{i=1}^{C}exp\left(F_{i}\cdot F_{j}\right)}F_{j}\right)+F_{j} \end{array}$$ (10)

Where, $F\in R^{C\times H\times W}$. *γ* is initialized as 0 and gradually learns to assign more weight. The final feature of each channel is the result of the weighted sum of the features of all channels and the original features, which models the long-term semantic dependencies between feature maps. The identifiability of features is enhanced, and the feature representation of changing remote-sensing building areas is highlighted.

Considering that low-level and high-level target buildings have differences in spatial distribution and physical appearance, it is challenging to characterize low-level target buildings effectively. The most common way to use low-level features is to connect low-level features with high-level features. However, this method can slightly improve the performance; it still uses a lot of irrelevant information in the process of semantic information aggregation, which reduces the semantic representation. To better utilize low-level features, we design an attention aggregation module with a residual strategy (RASAM) to enrich their semantic information; this operation bridges high-level and low-level features without sacrificing the low-level spatial details gap. It is worth noting that this method we designed mainly embeds high-level local attention into low-level features, which embed contextual information beyond the limits of their receptive fields while preserving their spatial details. Furthermore, to avoid excessive interference with high-level features, we add a residual design to emphasize the importance of low-level features. The enhanced underlying features are shown in equation.

$$f_{X_{A}^{l}}^{\prime s}=f_{X_{A}^{l}}^{s}+f_{X_{A}^{l}}^{s}\cdot \alpha_{l}$$ (11)

$$f_{X_{A}^{l}}^{s}=(f_{X_{A}}^{\prime s-1}+f_{X_{A}}^{s-1,c})$$ (12)

Where, *α*_*l*_ represent attention map coefficients. *α*_*l*_ as shown in equation.

$$\begin{array}{c} \alpha_{l}=F_{U}^{↑}\left(\sigma_{1}\left(H_{c}\sigma_{2}\left(F_{r}^{↓}\cdot \frac{1}{H^{u}W^{v}}\sum_{i=1}^{H_{u}}\sum_{j=1}^{W_{v}}\Theta_{c}\left(i,j\right)\right)\right)\right) \end{array}$$ (13)

where, $F_{r}^{↓}$ is a dimension reduction convolution and *H*_*c*_ changes the number of channels to be the same as $f_{X_{A}^{l}}^{s}$. *σ*_1_, *σ*_2_ denote sigmoid and ReLU functions, respectively; $F_{r}^{↓}$ represents the 1 × 1 dimension-reduction convolution with the reduction ratio *r*.

Through CPMA and RASAM, we get the final feature pair $F_{X_{A}}$ and $F_{X_{B}}$, as shown in the equation.

$$\begin{array}{c} f_{X_{A}}^{s,c}=Conv_{1\times 1}\left(f_{X_{A}}^{\prime s-1}⊕F_{U}^{↑}\left(f_{X_{A}}^{\prime s}\right)⊕f_{X_{A}^{l}}^{\prime s}\right) \end{array}$$ (14)

Where, $F_{U}^{↑}\left(\cdot \right)$ represents the upsampling operation of bilinear interpolation. In the 4th feature extraction stage, aggregate to form feature pair $F_{X_{A}}$ and $F_{X_{B}}$.

The classification module (CLM) mainly decodes the $F_{X_{A}}$ and $F_{X_{B}}$ of the features obtained through the Siamese network and realizes the precise segmentation of the changed and unchanged building areas in the remote sensing image. We use a set of continuous 3 × 3 convolutions and 1 × 1 convolutions as an essential part of the classification module.

### Weighted loss function

To obtain the optimal feature representation and avoid the network falling into a local optimal state during the feature extraction process, we design a weighted loss function, using cross-entropy to supervise each feature extraction stage separately and use the share loss. The function performs supervised learning on the entire network. The weighted loss function is calculated as shown in the equation.

$$\begin{array}{c} L_{Total}=\lambda \left(\sum_{s=1}^{S}\left(L_{s}\right)\right)+L_{d} \end{array}$$ (15)

Where, λ = 0.2 represents the learnable weight factor. *L*_*s*_ represents the cross-entropy loss of each stages, and *S* represents different feature extraction stages, namely, *S* = {1, 2, 3, 4}. *L*_*d*_ indicates dice loss. The calculation of *L*_*s*_ and *L*_*d*_ is shown in the equation.

$$L_{s}=\frac{1}{H\times W}\sum_{\{i,j\}=1}^{H,W}log\left(O_{i,j}^{\prime },Y_{i,j}\right)$$ (16)

$$L_{d}=1-\frac{2\cdot |O^{\prime }⋂Y|}{O^{\prime }⋃Y}$$ (17)

Where, *H*, *W* represent the height and width of the input image, respectively. *i*, *j* represents the pixel position. *O*′ indicates the predicted label. *Y* represents the ground truth label. Algorithm 1 shows the training process of the proposed SAASNets with building change detection framework.

## Experimental results and discussion

In this section, we first provide data sources, initialization parameter settings, and related evaluation indicators. Second, comparison results with state-of-the-art building change detection methods and internal structure ablation experiments of the proposed SAASNets framework are provided. Finally, examples are shown, and a corresponding discussion is given.

**Algorithm 1:** The buildings of remote sensing images change detection process by our develop SAASNets.

**Input:** Given a set of remote sensing building image pairs $X=\{X_{A},X_{B}|R^{H\times W\times C}\}$. H, W, and C represent the height, width, and channel dimensions, respectively. Weighted reconstruction loss function *L*_*Total*_ with a maximum number of iterations of *MaxEpochs*.

**for** *epochs = 0 to epochs = MaxEpochs* **do**

 $\left\{f_{X_{A}}^{s},f_{X_{B}}^{s}\right\}\overset{LAFEM}{\leftarrow }\left\{f_{X_{A}^{0}},f_{X_{B}}^{0}\right\}\overset{Eq.(1)}{\leftarrow }X\in R^{H\times W\times C}$;

 $\left\{f_{X_{A}}^{\prime s},f_{X_{B}}^{\prime s}\right\}\overset{CPMA}{\leftarrow }\left\{f_{X_{A}}^{s},f_{X_{B}}^{s}\right\}$;

 $\left\{f_{X_{A}^{l}}^{s},f_{X_{B}^{l}}^{s}\right\}\overset{RASAM}{\leftarrow }\left\{f_{X_{A}}^{\prime s},f_{X_{B}}^{\prime s}\right\}$;

 $\left\{F_{X_{A}},F_{X_{B}}\right\}\overset{Conv_{1\times 1}\left(\cdot \right)}{\leftarrow }\left\{f_{X_{A}^{l}}^{s},f_{X_{B}^{l}}^{s}\right\}$;

 The output of SAASNets is used for classification of unchange and change piexl;

**end**

**output:** optimization the training during by *AdamW* and loss function of *L*_*Total*_.

### Data and metrics

#### Data preparation

*LEVIR-CD*. [21, 34] This dataset consists of 637 0.5-meter-resolution remote sensing images with a time of 5 to 14 years, including various architectural changes such as villas, tall apartments, small garages, and large warehouses. For each pair of images, the size is 1024 × 1024. There are 445 pairs of training samples, 64 pairs of verification samples, and 128 pairs of test samples.

*CDD*. [35] The change detection dataset comprises 16,000 pairs of remote sensing images with different resolutions. Each image size is 256 × 256. Ten thousand pairs are used as training samples, 1,000 as verification samples, and 5,000 as test samples. To ensure the fairness and consistency of the experiment, in all methods, we use a series of image enhancement processes such as noise addition, color transformation, and flipping on the training samples of the two sets of baseline datasets.

#### Evaluation metrics

Overall Accuracy (OA), mean intersection-Union ratio (mIOU), and composite index F1 value are evaluation metrics for all methods. These metrics are calculated as shown in the equation.

$$\begin{array}{c} OA=\frac{TP+TN}{TP+TN+FP+FN} \end{array}$$ (18)

$$\begin{array}{c} F1=\frac{2\cdot TP}{2\cdot TP+FN+FP} \end{array}$$ (19)

$$\begin{array}{c} mIOU=\frac{1}{N}\sum_{n=1}^{N}\frac{P\cap G}{P\cup G} \end{array}$$ (20)

Where, *TP*, *TN*, *FP* and *FN* indicate true positive, true negative, false positive, false negative, respectively. *P* represents the predicted value, *G* represents the actual value, and *N* represents the total number of categories.

#### Parameter settings

In the training phase of the proposed SAASNets architectural change detection framework, we set the learning rate to 0.00025, the batch size to 16, and the training times to 100. To prevent overfitting, we enabled the early stop setting. The training will be stopped when it reaches 20 rounds if the loss value rises sharply or tends to 0. AdamW is used as the optimizer, and the decay rate is 0.095.

All the experiment development is completed based on python3.7.6 of ubuntu20, and the deep learning library is torch1.7.0+cuda11.0 and Numpy1.19.5, etc. At the same time, training and testing are carried out on four RTXA6000 GPUs.

### Comparison with state-of-the-art methods

To demonstrate the effectiveness of the proposed SAASNets multispectral remote sensing with building change detection framework. We compare state-of-the-art change detection methods on two baseline datasets and give corresponding experimental results and visual demonstrations. The experimental results of different methods are shown in Tables 1 and 2.

**Table 1:** Experimental results of different methods on the CDD datasets. SAASNets indicates our proposed change detection networks.

	OA(%)	mIOU(%)	*F*_1_(%)
FTN	92.71	84.97	89.56
SARASNet	92.74	84.9	89.52
TINYCD	92.95	85.94	90.12
DDPM	92.83	85.22	89.7
EGDE-Net [36]	93.12	86.78	90.57
SAAN [37]	93.35	87.15	91.05
SAASNets	93.44	87.96	91.28

**Table 2:** Experimental results of different methods on the LEVIR-CD datasets. SAASNets indicates our proposed change detection networks.

	OA(%)	mIOU(%)	*F*_1_(%)
FTN	93.88	84.86	89.42
SARASNet	93.99	85.5	89.8
TINYCD	94.07	86.28	90.27
DDPM	94.01	85.73	89.95
EGDE-Net [36]	94.11	86.59	90.32
SAAN [37]	94.16	87.04	90.37
SAASNets	94.18	87.11	90.76

According to Tables 1 and 2, we can draw the following conclusions:

1. Our proposed multispectral remote sensing architectural change detection framework of SAASNets achieves state-of-the-art performance on both baseline datasets. For example, the indicators on the CDD data set are 93.44%, 87.96%, and 91.28%, respectively. On the LEVIR-CD dataset, the three indicators are 94.18%, 87.11%, and 90.76%, respectively. The main reasons are twofold: on the one hand, embedding attention in the subspace of each feature extraction component refines the local and global spatial information and improves the representation performance of features. On the other hand, the fusion stage uses channel and position multi-head attention modules to promote the interaction between local and global features and supervises and adjusts each feature extraction stage through a weighted loss function, further improving the network perception of subtle changes in architectural objects, leading to the optimal performance of the proposed framework.
2. The FTN method achieved the worst performance on the LEVIR-CD dataset, while the SARASNet method achieved the worst performance on the CDD dataset. The FTN method is 0.38% lower than the SARASNet method on the LEVIR-CD dataset, but it is increased by 0.04% on the CDD dataset. Compared with the SARASNet method, the FTN method uses SWin-Transformer as a feature extractor to expand the feature map. The receptive field is more sensitive to buildings with large-scale changes and combines multi-level visual features in a pyramid manner. In contrast, the SARASNet method combines multi-level features through cross-transformers and pays more attention to the spatial information of architectural targets in remote sensing images.
3. Compared with the DDPM method, the TINYCD method has better detection performance on the two data sets. For example, mIOU increased by 0.72% and 0.55%, respectively. The TINYCD method uses a pixel-level mask generator (PW-MLP) to process complex semantic information and generates a score as spatiotemporal attention and a mask tensor. A weighting operation is performed on each pixel to alleviate the misleading information generated by up-sampling, thereby improving the detection accuracy of changing buildings in multispectral remote sensing images.

**Fig 2:**
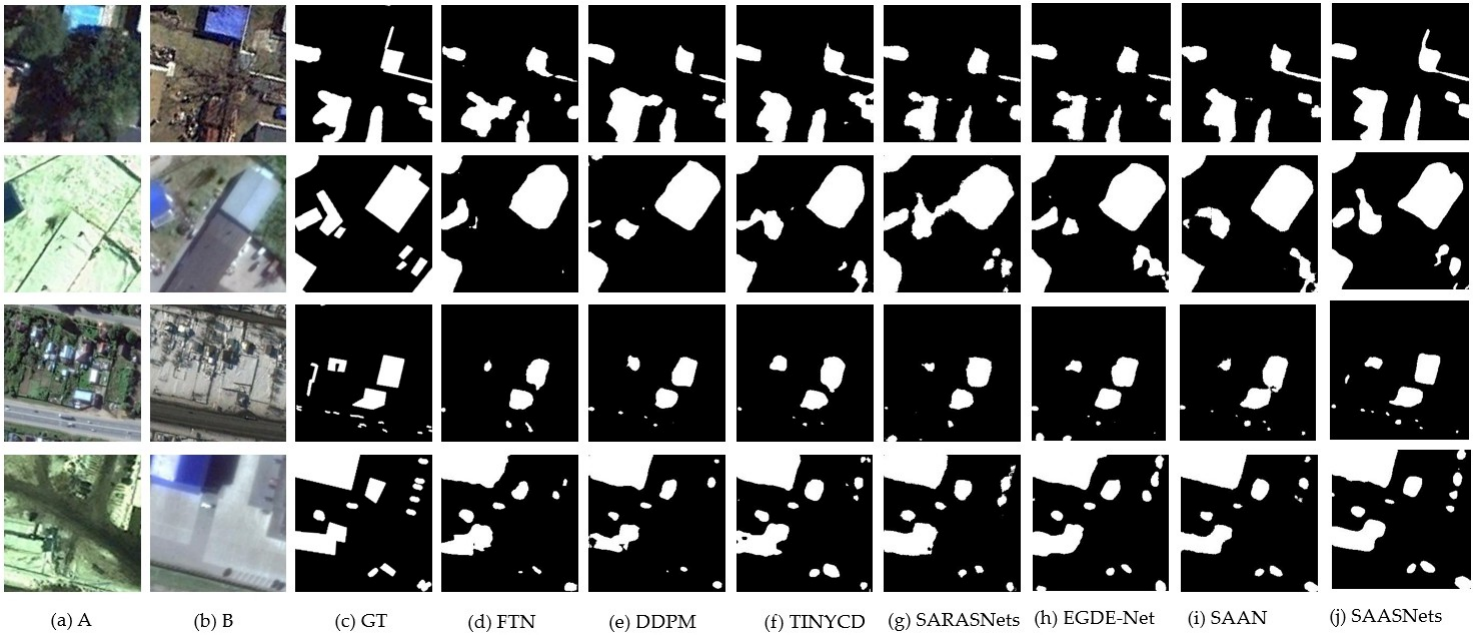
Visual demonstration of different methods on the CCD dataset. Where, (a) to (c) represent the images before and after the change and ground truth labels, respectively. (d) to (i) represent the detection results of different methods. (j) indicates our proposed SAASNets framework.

**Fig 3:**
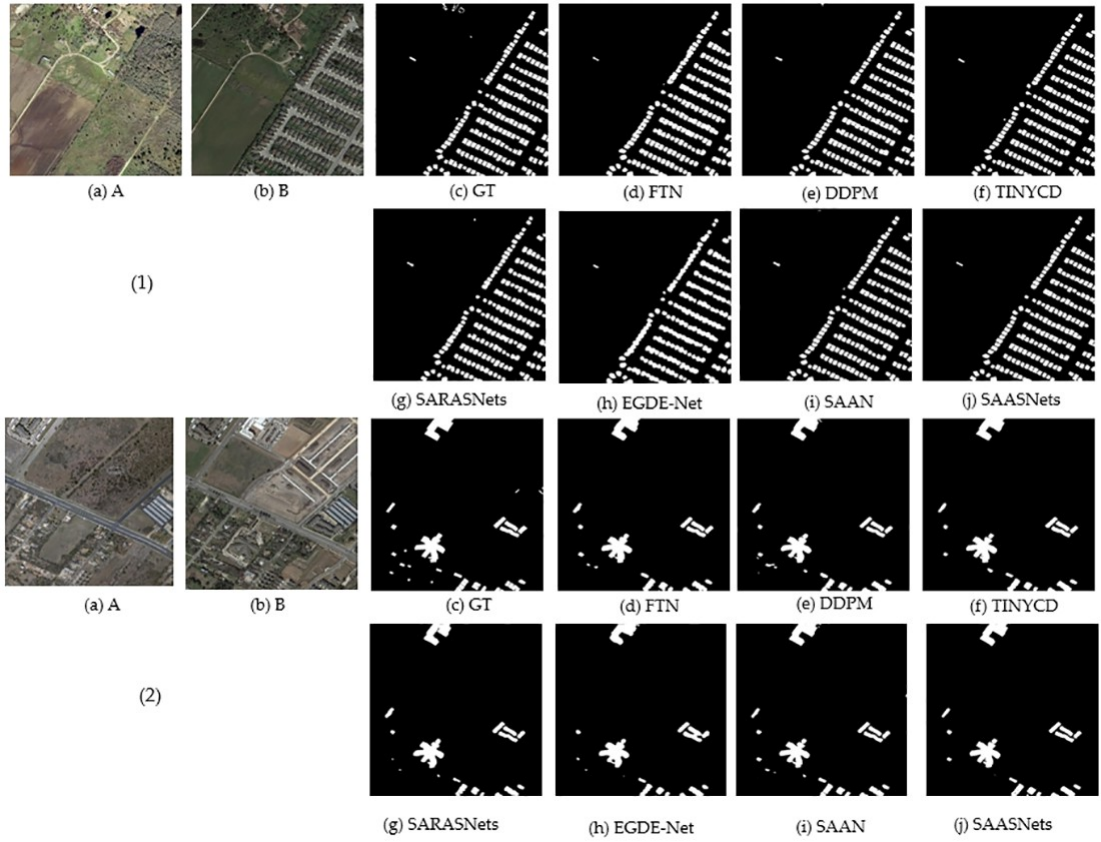
Visual demonstration of different methods on the LEVIR-CD dataset. Where, (a) to (c) represent the images before and after the change and ground truth labels, respectively. (d) to (i) represent the detection results of different methods. (j) indicates our proposed SAASNets framework.

In addition, in the LEVIR-CD and CDD datasets, EGDE-Net and SAAN change detection algorithms have achieved good competitive advantages. For example, the SAAN method of F1 on CDD increased by 1.35% and 0.93% for the DDPM and TINYCD methods, respectively. The mIOU of EGDE-Net increased by 1.31% compared with the DDPM method. The SAAN method similarly flows attention maps, enhances the representation of changing features, suppresses irrelevant non-changing information, solves the problem of inconsistent feature similarity in different stages, and realizes the flow of similar relationships in different decoding stages. This maintains the model’s perception of feature similarity relationships. The EGDE-Net method encodes the dual-branch network through an edge-guided Transformer module, refining the remote context and edge features. At the same time, it uses the feature difference enhancement module to learn the difference between dual-phase features. This improves the accuracy of transform detection. A visual demonstration of the different methods is shown in Figs 2 and 3. It can also be seen from Figs 2 and 3 that the demonstration effect of our proposed SAASNets detection framework on the CDD and LEVIR-CD datasets is optimal. Namely, the acquisition of local details and global semantics is also better, and at the same time, the demonstration effects of SAAN and EGDE also have obvious advantages.

### Ablation study

To prove whether each component in the proposed SAASNets multispectral remote sensing building change detection framework plays an active role in the overall network, we evaluate and verify each component on the LEVIR-CD and CDD datasets and give the experimental results and corresponding analysis. The experimental results of different modules are shown in Tables 3 and 4.

**Table 3:** Experimental results of different methods on the CDD datasets. SAASNets indicates our proposed change detection networks. Res2net50 indicates the initial backbone network. CPMA indicates channel position multi-head attention module. LAFEM indicates feature extraction module with special attention embedding. RASAM indicates the attention feature fusion module with residual strategy. *L*_*d*_ indicates the dice loss function.

	OA(%)	mIOU(%)	*F*_1_(%)
Res2net50+CPMA	92.66	84.72	89.41
LAFEM+CPMA	92.94	85.68	89.97
LAFEM+CPMA+RASAM+*L*_*d*_	93.4	87.79	91.18
SAASNets	93.44	87.96	91.28

**Table 4:** Experimental results of different methods on the LEVIR-CD datasets. SAASNets indicates our proposed change detection networks. Res2net50 indicates the initial backbone network. CPMA indicates channel position multi-head attention module. LAFEM indicates feature extraction module with special attention embedding. RASAM indicates the attention feature fusion module with residual strategy. *L*_*d*_ indicates the dice loss function.

	OA(%)	mIOU(%)	*F*_1_(%)
Res2net50+CPMA	93.82	84.02	88.91
LAFEM+CPMA	93.89	84.91	89.45
LAFEM+CPMA+RASAM+*L*_*d*_	94.06	86.02	90.12
SAASNets	94.18	87.11	90.76

From Tables 3 and 4, we can draw the following conclusions:

1. The various modules in the proposed SAASNets framework cooperate to promote the optimal detection performance of the network. That is, each module contributes positively to the overall network. For example, on the LEVIR-CD dataset, the OA, mIOU, and F1 of SAASNets are 0.12%, 1.09%, and 0.64% higher than those of LAFEM+CPMA+RASAM+*L*_*d*_, respectively. The improvements on the CDD dataset are 0.04%, 0.17%, and 0.1%. This demonstrates that our designed weighted loss function, which supervises each feature extraction stage individually, is beneficial for representing architectural features in multispectral remote-sensing images.
2. Compared with the Res2net50+CPMA building change detection method, the LAFEM+CPMA method improves the three indicators on the LEVIR-CD dataset by 0.07%, 0.89%, and 0.54%, respectively. The improvements on the CDD dataset are 0.28%, 0.86%, and 0.56%. This shows that we embed a particular attention module in each subspace of the initial multi-scale feature extractor, which is beneficial to the representation of local details and can effectively aggregate contextual global semantics. At the same time, the relationship between global and local semantics is improved during the aggregation process. The interaction among them improves the description of architectural details through multi-scale features.
3. On the LEVIR-CD and CDD datasets, LAFEM+CPMA+RASAM+*L*_*d*_ have achieved better competitiveness, such as F1 increased by 1.21% and 0.67% compared with LAFEM+CPMA, respectively. This proves the proposed RASAM module can effectively aggregate Siamese features and screen redundant information.

### Examples and discussion

To further demonstrate the effectiveness of the proposed SAASNets approach, we provide visualizations of validation losses and associated images for different building change detection methods.

#### Validation of valid losses

**Fig 4:**
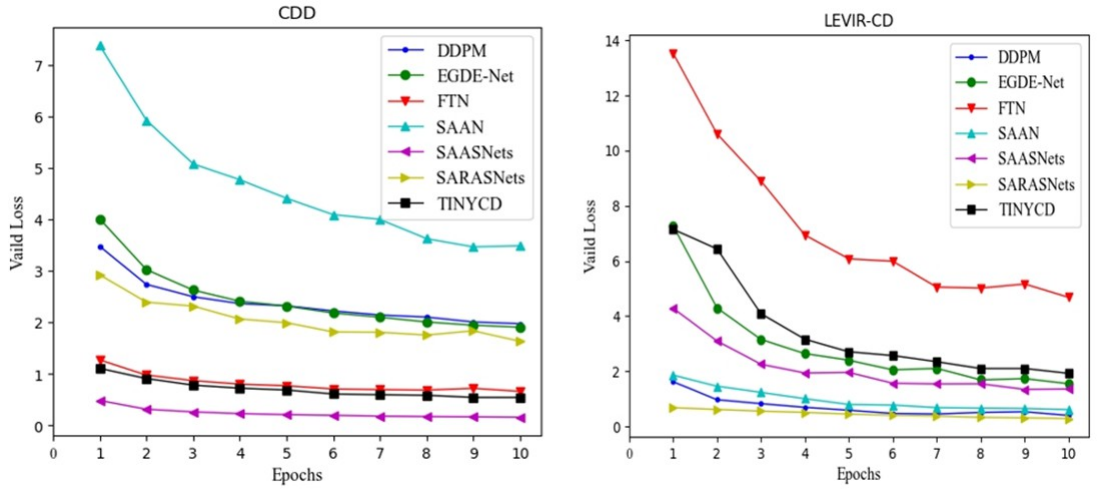
Demonstration of validation loss convergence for different methods. Among them, CDD and LEVIR-CD represent different baseline datasets.

The convergence of the validation loss is shown in Fig 4.

It can be seen from Fig 4 that in the proposed SAASNets architectural change detection framework, the verification loss on the CDD and LEVIR-CD datasets has been declining. The faster convergence speed in the first four times may be due to the significant learning settings. After that, the convergence is relatively stable. It is worth noting that there are slight fluctuations in the LEVIR-CD dataset. At the same time, the initial validation loss value is immense, which may be caused by significant changes in building scales in the image. Tables 5 and 6 give the impact of different λ on the overall performance of our proposed SAASNets transformation detection framework.

**Table 5:** Experimental results of different λ with *L*_*s*_ loss function on the CDD datasets. In our proposed SAASNets detection framework, λ = 0.2.

	OA(%)	mIOU(%)	*F*_1_(%)
λ = 0.1	93.27	87.93	91.25
λ = 0.3	93.15	87.58	90.99
λ = 0.4	93.07	87.42	90.25
λ = 0.5	93.01	87.05	90.14

**Table 6:** Experimental results of different λ with *L*_*s*_ loss function on the LEVIR-CD datasets. In our proposed SAASNets detection framework, λ = 0.2.

	OA(%)	mIOU(%)	*F*_1_(%)
λ = 0.1	94.11	87.07	90.53
λ = 0.3	93.97	86.85	90.20
λ = 0.4	93.84	86.71	90.04
λ = 0.5	93.09	86.55	89.92

Tables 5 and 6 show that when λ > 0.2, as c increases, the detection accuracy of the proposed framework on both data sets decreases. When λ = 0.1, the proposed framework has good competitive advantages. As λ increases, the feature extractor may get stuck in a local optimal state and need help to obtain the best feature representation.

#### Complexity of different methods

**Fig 5:**
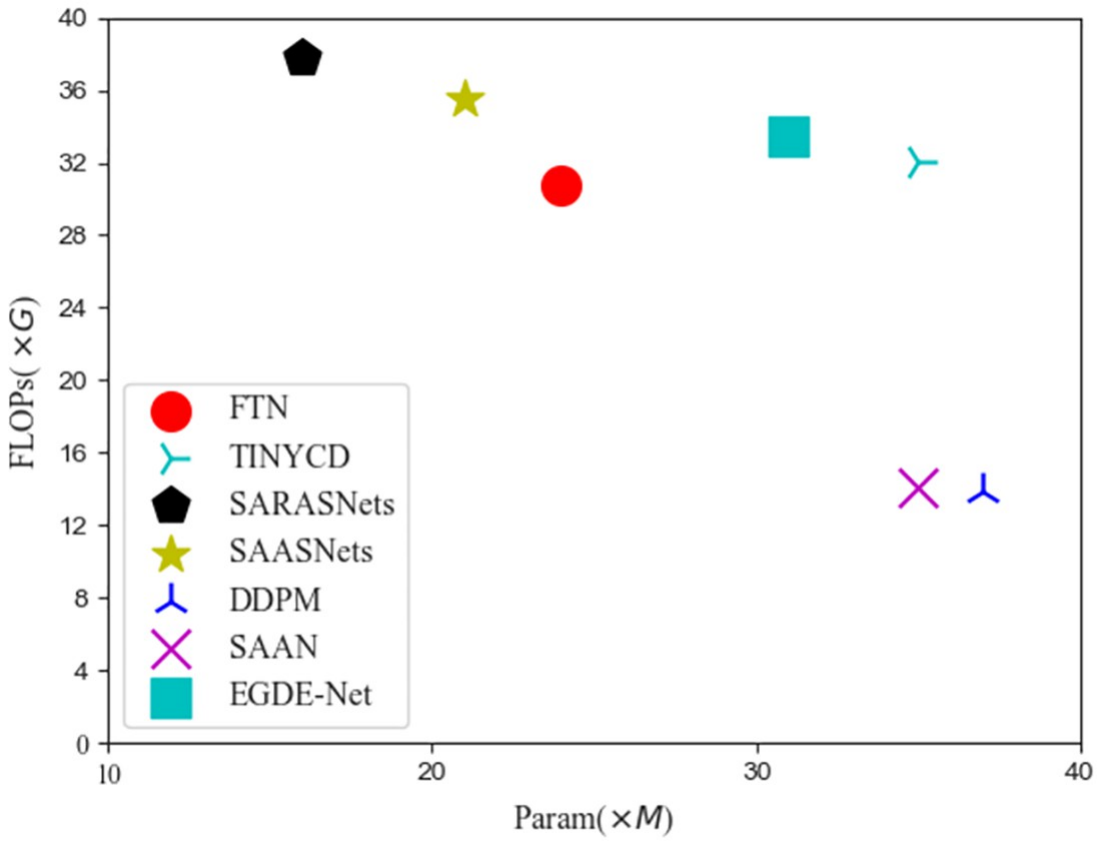
Demonstration of validation loss convergence for different methods. Among them, CDD and LEVIR-CD represent different baseline datasets.

Fig 5 provides the change detection efficiency of different methods for building targets in multispectral remote sensing images.

According to Fig 5, our proposed SAASNets building change detection method still has good detection efficiency while ensuring accuracy. Although the detection efficiency is worse than the SARASNets method, our detection accuracy is better than this method. Our proposed method is significantly superior in accuracy and efficiency compared with other building change detection methods. For example, SAAN and TINYCD have similar parameter amounts (Param), but the detection efficiency of TINYCD is significantly better than SAAN. Namely, TINYCD of FLOPs is 18.0G higher than TINYCD. The segmentation efficiency and parameter amount (Param) of the SARASNets method are better than the SAASNets method we proposed, but the detection accuracy changes are poor.

### Discussion

**Fig 6:**
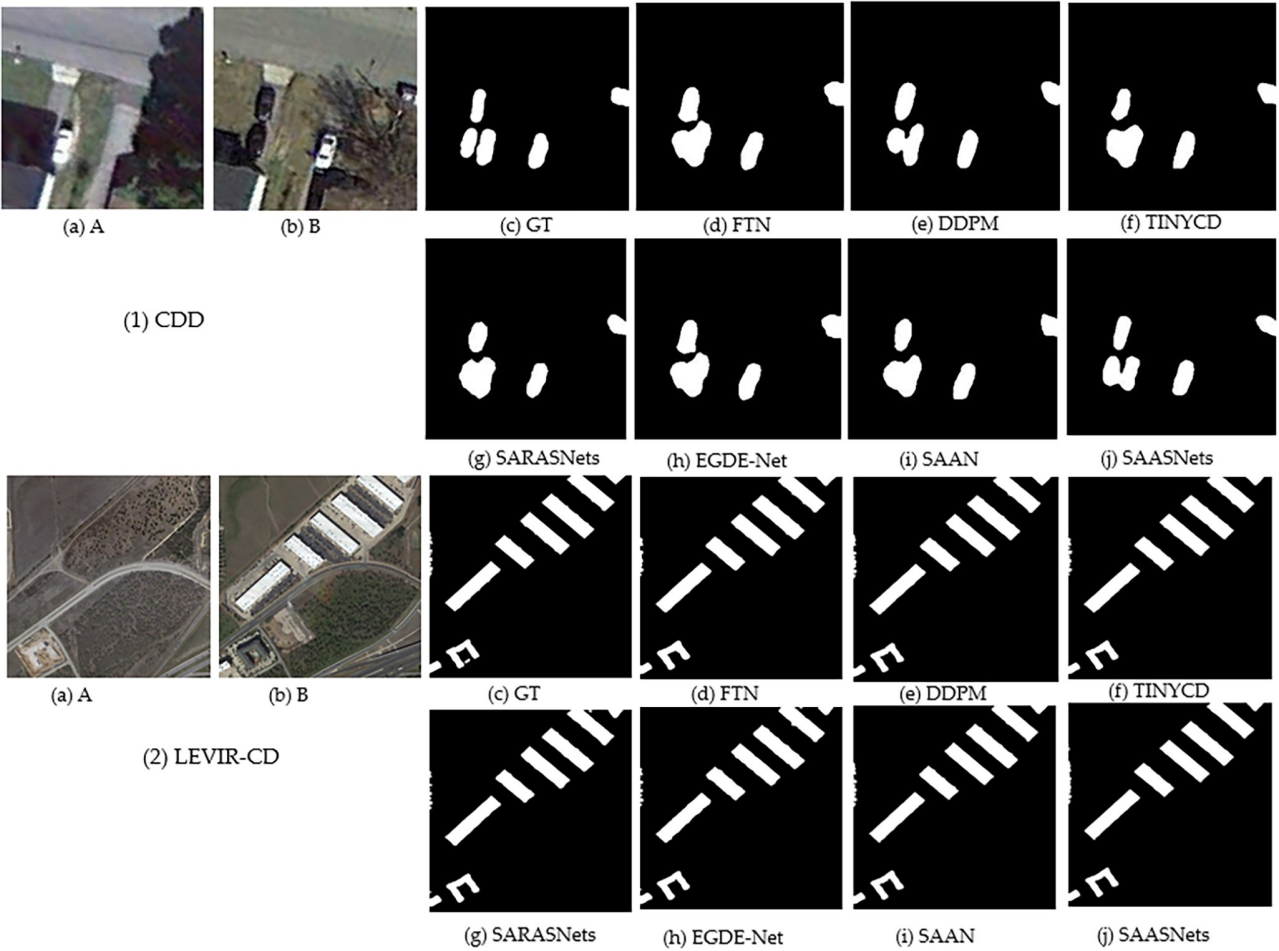
Different methods for edge visualization on different datasets. Where, (1) indicates the CDD datasets. (2) indicates the LEVIR-CD datasets. (a) indicates the building image before the change. (b) indicates the building image after the change. (c) represent the ground truth labels of change (d) FIN to (i) SAAN represent the detection results of different methods. (j) indicates the proposed SAASNets building change detection framework.

To visually demonstrate the excellent detection performance of our proposed SAASNets building change detection method, Fig 6 shows the visualization results of different methods on the CDD and LEVIR-CD baseline datasets.

From Fig 6, we can also clearly find that our proposed SAASNets change detection framework performs better on building edges of different scales. The possible reason is that, on the one hand, we use a multi-scale strategy to conduct the description, and the information is refined by using a particular attention module, which effectively distinguishes the difference between the architectural change area and the peripheral organizational structure. On the other hand, the multi-head attention mechanism using channels and positions reduces the use of redundant information, and the attention module using the residual strategy establishes dependencies and interactions between local and global features, prompting the network to acquire better edge information.

## Conclusions and next plans

This paper considers that the traditional multispectral remote sensing building change detection method based on a convolutional neural network is limited by the receptive field, and it is challenging to obtain the detailed semantics of buildings in the image entirely. A Siamese Network of Shared Attention Aggregation (SAASNets) is proposed, which embeds attention modules in each subspace of the feature extraction stage, reduces the use of redundant information, and combines attention features and convolution features to improve local features and the expressive power of global context semantics. In addition, to form an interaction between Siamese local features and global features, channel and position multi-head attention modules are adopted to establish correlations between channel information and long-term dependencies between position information to highlight the representation of salient features while improving the accuracy of building change detection. Finally, the evaluation and demonstration of various open-source datasets show that the proposed framework has better accuracy and robustness.

During the training process, the parameter setting of the proposed method could be more convenient, and there is still a lot of room for improvement in detection efficiency. Therefore, in the subsequent work, we will proceed from the above two aspects to design a concise and efficient semantic guidance network, which can further improve the accuracy of building change detection while reducing parameters and improving efficiency.

## References

[1] Kushwaha Naveen Kumar, Chaudhuri Debasis, and Singh Manish Pratap. “Automatic Bright Circular Type Oil Tank Detection Using Remote Sensing Images.” Defence Science Journal 63.3 (2013). doi: 10.14429/dsj.63.2737

[2] Li Jun, et al. “A review of remote sensing for environmental monitoring in China.” Remote Sensing 12.7 (2020): 1130.

[3] Lisowski Józef. “Artificial intelligence methods in safe ship control based on marine environment remote sensing.” Remote Sensing 15.1 (2022): 203. doi: 10.3390/rs15010203

[4] Goswami Anjali, et al. “Change detection in remote sensing image data comparing algebraic and machine learning methods.” Electronics 11.3 (2022): 431.

[5] Zhu W, Chen J, Sun Q, et al. Reconstructing of high-spatial-resolution three-dimensional electron density by ingesting SAR-derived VTEC into IRI model[J]. IEEE Geoscience and Remote Sensing Letters, 2022, 19: 1–5. doi: 10.1109/LGRS.2021.3139774

[6] Zhou G, Zhang H, Xu C, et al. A Real-Time Data Acquisition System for Single-Band Bathymetric LiDAR[J]. IEEE Transactions on Geoscience and Remote Sensing, 2023.

[7] Zhou G, Zhao D, Zhou X, et al. An RF amplifier circuit for enhancement of echo signal detection in bathymetric LiDAR[J]. IEEE Sensors Journal, 2022, 22(21): 20612–20625. doi: 10.1109/JSEN.2022.3206763

[8] Ji Shunping, et al. “Building instance change detection from large-scale aerial images using convolutional neural networks and simulated samples.” Remote Sensing 11.11 (2019): 1343.

[9] Mohammadian Amir, and Ghaderi Foad. “SiamixFormer: a fully-transformer Siamese network with temporal Fusion for accurate building detection and change detection in bi-temporal remote sensing images.” International Journal of Remote Sensing 44.12 (2023): 3660–3678. doi: 10.1080/01431161.2023.2225228

[10] Chen Hongruixuan, et al. “Change detection in multisource VHR images via deep siamese convolutional multiple-layers recurrent neural network.” IEEE Transactions on Geoscience and Remote Sensing 58.4 (2019): 2848–2864.

[11] Zheng Zhi, et al. “CLNet: Cross-layer convolutional neural network for change detection in optical remote sensing imagery.” ISPRS Journal of Photogrammetry and Remote Sensing 175 (2021): 247–267. doi: 10.1016/j.isprsjprs.2021.03.005

[12] Papadomanolaki Maria, Vakalopoulou Maria, and Karantzalos Konstantinos. “A deep multitask learning framework coupling semantic segmentation and fully convolutional LSTM networks for urban change detection.” IEEE Transactions on Geoscience and Remote Sensing 59.9 (2021): 7651–7668. doi: 10.1109/TGRS.2021.3055584

[13] Saha Sudipan, Bovolo Francesca, and Bruzzone Lorenzo. “Change detection in image time-series using unsupervised lstm.” IEEE Geoscience and Remote Sensing Letters 19 (2020): 1–5.

[14] Zhang, Lan, Bo Zhong, and Aixia Yang. “Building change detection using object-oriented LBP feature map in very high spatial resolution imagery.” 2019 10th International Workshop on the Analysis of Multitemporal Remote Sensing Images (MultiTemp). IEEE, 2019.

[15] Xi Yicheng, and Luo Qingli. “A morphology-based method for building change detection using multi-temporal airborne LiDAR data.” Remote Sensing Letters 9.2 (2018): 131–139. doi: 10.1080/2150704X.2017.1402384

[16] Wang Haibo, et al. “A refined method of high-resolution remote sensing change detection based on machine learning for newly constructed building areas.” Remote Sensing 13.8 (2021): 1507.

[17] Zong Kaibin, Sowmya Arcot, and Trinder John. “Building change detection from remotely sensed images based on spatial domain analysis and Markov random field.” Journal of Applied Remote Sensing 13.2 (2019): 024514–024514.

[18] Saha Sudipan, Bovolo Francesca, and Bruzzone Lorenzo. “Building change detection in VHR SAR images via unsupervised deep transcoding.” IEEE Transactions on Geoscience and Remote Sensing 59.3 (2020): 1917–1929.

[19] Sun Ying, et al. “Fine-grained building change detection from very high-spatial-resolution remote sensing images based on deep multitask learning.” IEEE Geoscience and Remote Sensing Letters 19 (2020): 1–5.

[20] Jiang Huiwei, et al. “PGA-SiamNet: Pyramid feature-based attention-guided Siamese network for remote sensing orthoimagery building change detection.” Remote Sensing 12.3 (2020): 484. doi: 10.3390/rs12030484

[21] Chen Hao, Li Wenyuan, and Shi Zhenwei. “Adversarial instance augmentation for building change detection in remote sensing images.” IEEE Transactions on Geoscience and Remote Sensing 60 (2021): 1–16. doi: 10.1109/TGRS.2020.3034752

[22] Zheng Hanhong, et al. “HFA-Net: High frequency attention siamese network for building change detection in VHR remote sensing images.” Pattern Recognition 129 (2022): 108717. doi: 10.1016/j.patcog.2022.108717

[23] Song Kaiqiang, and Jiang Jie. “AGCDetNet: An attention-guided network for building change detection in high-resolution remote sensing images.” IEEE Journal of Selected Topics in Applied Earth Observations and Remote Sensing 14 (2021): 4816–4831. doi: 10.1109/JSTARS.2021.3077545

[24] Bai Beifang, et al. “Edge-guided recurrent convolutional neural network for multitemporal remote sensing image building change detection.” IEEE Transactions on Geoscience and Remote Sensing 60 (2021): 1–13.

[25] Xue Junkang, et al. “Multi-feature enhanced building change detection based on semantic information guidance.” Remote Sensing 13.20 (2021): 4171.

[26] Liu Mengxi, et al. “PA-Former: learning prior-aware transformer for remote sensing building change detection.” IEEE Geoscience and Remote Sensing Letters 19 (2022): 1–5. doi: 10.1109/LGRS.2022.3226859

[27] Zheng Jiaxiang, et al. “MDESNet: Multitask Difference-Enhanced Siamese Network for Building Change Detection in High-Resolution Remote Sensing Images.” Remote Sensing 14.15 (2022): 3775.

[28] Li X, Li Y, Ai J, et al. Semantic segmentation of UAV remote sensing images based on edge feature fusing and multi-level upsampling integrated with Deeplabv3+[J]. Plos one, 2023, 18(1): e0279097. doi: 10.1371/journal.pone.0279097 36662763 PMC9858408

[29] Coupland K, Hamilton D, Griess V C. Combining aerial photos and LiDAR data to detect canopy cover change in urban forests[J]. Plos one, 2022, 17(9): e0273487. doi: 10.1371/journal.pone.0273487 36103468 PMC9473407

[30] Xu Chuan, et al. “SCAD: A Siamese Cross-Attention Discrimination Network for Bitemporal Building Change Detection.” Remote Sensing 14.24 (2022): 6213. doi: 10.3390/rs14246213

[31] Zhou Yong, et al. “Spatial-Temporal Based Multihead Self-Attention for Remote Sensing Image Change Detection.” IEEE Transactions on Circuits and Systems for Video Technology 32.10 (2022): 6615–6626. doi: 10.1109/TCSVT.2022.3176055

[32] Hu Meiqi, Wu Chen, and Zhang Liangpei. “GlobalMind: Global Multi-head Interactive Self-attention Network for Hyperspectral Change Detection.” arXiv preprint arXiv:2304.08687 (2023).

[33] Zhang Qijian, et al. “Dense attention fluid network for salient object detection in optical remote sensing images.” IEEE Transactions on Image Processing 30 (2020): 1305–1317. 33306467 10.1109/TIP.2020.3042084

[34] Xia Liegang, et al. “Building change detection based on an edge-guided convolutional neural network combined with a transformer.” Remote Sensing 14.18 (2022): 4524.

[35] Lebedev M. A., et al. “Change detection in remote sensing images using conditional adversarial networks.” The International Archives of the Photogrammetry, Remote Sensing and Spatial Information Sciences 42 (2018): 565–571.

[36] Chen Z, Zhou Y, Wang B, et al. EGDE-Net: A building change detection method for high-resolution remote sensing imagery based on edge guidance and differential enhancement[J]. ISPRS Journal of Photogrammetry and Remote Sensing, 2022, 191: 203–222. doi: 10.1016/j.isprsjprs.2022.07.016

[37] Guo H, Su X, Wu C, et al. SAAN: Similarity-aware attention flow network for change detection with VHR remote sensing images[J]. arXiv preprint arXiv:2308.14570, 2023. 10.1109/TIP.2024.3349868 38427550

